# Asymmetrical atrophy of thalamic subnuclei in Alzheimer's disease and amyloid-positive mild cognitive impairment is associated with key clinical features

**DOI:** 10.1016/j.dadm.2019.08.001

**Published:** 2019-10-01

**Authors:** Audrey Low, Elijah Mak, Maura Malpetti, Leonidas Chouliaras, Nicolas Nicastro, Li Su, Negin Holland, Timothy Rittman, Patricia Vázquez Rodríguez, Luca Passamonti, W Richard Bevan-Jones, PP Simon Jones, James B. Rowe, John T. O'Brien

**Affiliations:** a Department of Psychiatry, University of Cambridge, Cambridge, United Kingdom; b Department of Clinical Neurosciences, University of Cambridge, Cambridge, United Kingdom

**Keywords:** Alzheimer's disease, Mild cognitive impairment, Cognitive aging, Thalamus, Magnetic resonance imaging

## Abstract

**Introduction:**

Although widespread cortical asymmetries have been identified in Alzheimer's disease (AD), thalamic asymmetries and their relevance to clinical severity in AD remain unclear.

**Methods:**

Lateralization indices were computed for individual thalamic subnuclei of 65 participants (33 healthy controls, 14 amyloid-positive patients with mild cognitive impairment, and 18 patients with AD dementia). We compared lateralization indices across diagnostic groups and correlated them with clinical measures.

**Results:**

Although overall asymmetry of the thalamus did not differ between groups, greater leftward lateralization of atrophy in the ventral nuclei was demonstrated in AD, compared with controls and amyloid-positive mild cognitive impairment. Increased posterior ventrolateral and ventromedial nuclei asymmetry were associated with worse cognitive dysfunction, informant-reported neuropsychiatric symptoms, and functional ability.

**Discussion:**

Leftward ventral thalamic atrophy was associated with disease severity in AD. Our findings suggest the clinically relevant involvement of thalamic nuclei in the pathophysiology of AD.

## 1 Introduction

Alzheimer's disease (AD) is the most common neurodegenerative disease worldwide, affecting more than 35 million people [1]. The cerebral atrophy characteristic of AD is bilateral but may not be symmetrical across hemispheres. With the advent of high-resolution magnetic resonance imaging (MRI), detailed brain atlases, probabilistic models of neuroanatomy, and advanced neuroimaging segmentation tools, there is a growing body of evidence that AD is asymmetrical [2], [3], [4], [5]. This has been supported by studies which have found that AD is associated with asymmetrical cortical thinning [2], cortical surface area [3], and global cerebral atrophy [4] across hemispheres.

The degree of cerebral asymmetry in AD has been associated with poorer cognitive outcomes [6], greater disease severity [7], and neuropsychiatric symptoms [8], [9]. In general, the asymmetrical cerebral involvement in AD is considered to be nondirectional, or as Derflinger and colleagues describe it, “asymmetric but not lateralized” [4], [5]. However, a number of studies have reported consistent left-lateralized damage in AD, particularly to the hippocampus and white matter integrity [10], [11], [12], [13], [14], and also in terms of amyloid and tau pathology [6], [15]. Furthermore, the degree of leftward cerebral asymmetry has been associated with disease progression and progressively declining cognitive performance [6], [10], [13], [14].

Atrophy in AD is asymmetrical across a wide range of brain structures, such as the medial temporal lobe [16], hippocampus [2], [5], [12], [17], amygdala [5], and caudate [5]. Asymmetries have also been found in terms of white matter integrity, neural connectivity [11], [13], cerebral metabolism [6], [18], amyloid-β deposition [6], and tau pathology [15]. Amyloid and tau asymmetries are positively correlated across regions typically affected with AD pathology, suggesting that the two pathologies interact locally [19]. In addition, asymmetries in different cerebral structures have also been found to correlate with one another, which might suggest a common underlying pathology driving asymmetrical alterations [20].

Most studies assessing cerebral asymmetry in AD have largely focused on the hippocampus. However, structures in the Papez circuit have also been implicated in AD pathology [21]. One such structure of importance is the thalamus [22], known for its function as a primary relay station, with a crucial role in the coordination and integration of cerebral processes. Although the Braak staging of AD pathology heavily emphasizes hippocampal changes, marked neurofibrillary deposits in the thalamus have also been found at the same stage as the hippocampus (stages III–IV), ahead of changes in other regions [23].

Although the prevalence of asymmetrical cerebral alterations has been well established across the brain in AD, there are a few studies investigating asymmetries of the human thalamus. The thalamus is highly connected through thalamocortical projections, and cortical asymmetries in AD have been associated with asymmetries of the thalamus, suggesting that similar patterns of asymmetries should also be present in the thalami of patients with AD [20]. However, investigations examining the thalamus as a uniform structure have reported a lack of significant asymmetry [5], [24], [25].

We hypothesized that thalamic asymmetries in AD could be masked when examining the thalamus as a uniform structure and that asymmetries may be confined to specific subnuclei. Given the growing evidence that individual thalamic subnuclei are distinct in morphology, function, and neural connectivity, the information derived from individual subnuclei may demonstrate increased sensitivity to pathological changes in AD and clinical correlates. Understanding the asymmetry of brain atrophy provides useful disease-related information concerning disease aetiology, clinical measures, and disease progression [7], [17], [26], and measures of cerebral asymmetry could even be more accurate than absolute bihemispheric measurements in detecting some disorders [27]. Using a novel probabilistic atlas [28], we aimed to examine whether the extent of thalamic asymmetry varied at a subnucleic level between healthy controls (HC) and patients with mild cognitive impairment (MCI) or clinical AD and its associations with measures of clinical severity. Given the novelty of our investigation, this study used an exploratory approach to determine the clinical relevance of differential thalamic atrophy.

## 2 Methods

The study cohort comprised 65 participants (33 HC, 14 positron-emission tomography amyloid-positive patients with MCI, and 18 patients with AD dementia) above the age of 50 years as part of the Neuroinflammation in Memory and Related Other Disorders study, a multimodal imaging cohort study [29]. Participants with MCI and AD were recruited from memory clinics in and around Cambridgeshire, including the regions of Lincolnshire, Bedfordshire, Norfolk, Suffolk, Hertfordshire, and Essex, or via the Dementias and Neurodegeneration specialty of the UK Clinical Research Network or the Join Dementia Research platform (https://www.joindementiaresearch.nihr.ac.uk). Participants classified as having MCI did not meet the criteria for probable AD but had memory impairments beyond what is expected for their age and years of education not explained by another diagnosis and had a Mini-Mental State Examination (MMSE) score >24 [30]. Only amyloid-positive patients with MCI were included in this study, as defined by an average ^11^C-Pittsburgh Compound B standardized cortical/cerebellar uptake ratio of above 1.5. Probable AD was defined according to the National Institute on Aging-Alzheimer's Association diagnostic guidelines [31]. HC were recruited from the Dementias and Neurodegeneration specialty of the UK Clinical Research Network, Join Dementia Research platform, and among friends, partners, and spouses of patients. HC were required to have an MMSE score >26, in the absence of regular memory loss symptoms and symptoms suggestive of dementia or unstable/significant medical illness. All participants underwent detailed clinical and neuropsychological assessments. Global cognition was assessed using the MMSE and Addenbrooke's Cognitive Examination–Revised (ACE-R), episodic memory was assessed using the Rey Auditory Verbal Learning Test total score, and language was assessed using a combined verbal fluency score obtained from ACE-R subscales. For participants with MCI and AD, the ability to carry out everyday activities was measured using the informant-completed Bristol Activities of Daily Living Scale and neuropsychiatric symptoms were assessed using the Neuropsychiatric Inventory. The detailed procedures have been described in a previously published study [29].

### 2.1 Image acquisition and processing

Participants underwent T1-weighted brain MRI at the Wolfson Brain Imaging Centre using a magnetization prepared rapid gradient echo sequence (176 slices, 1.0 mm thickness, repetition time = 2300 ms, echo time = 2.98 ms, field of view = 256 × 240 mm^2^, flip angle = 9°, and voxel size = 1.0 × 1.0 × 1.0 mm³) on a Siemens 3T Tim Trio or Verio (Siemens Healthcare, Erlangen, Germany).

**Fig. 1:**
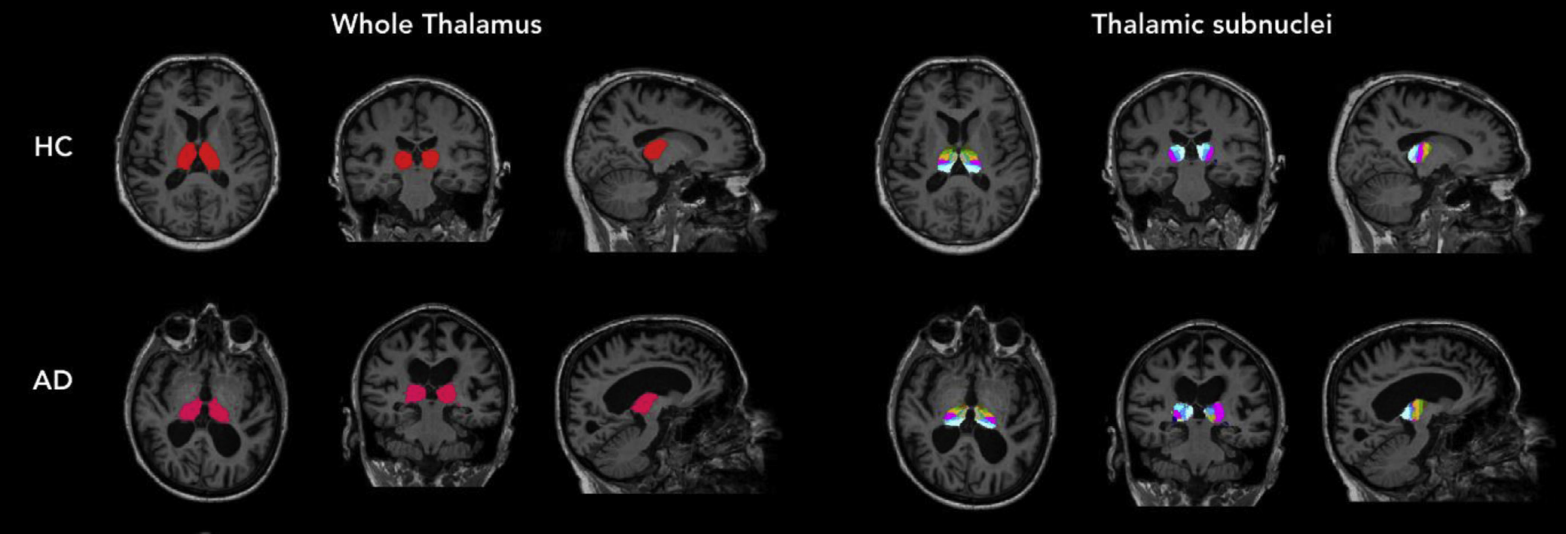
Segmentations of thalamic subnuclei in healthy controls (HC) and Alzheimer's disease (AD).

To segment the thalami into individual subnuclei, we used an automated segmentation tool in a developmental version of FreeSurfer 6.0 (accessed December 2018) (http://surfer.nmr.mgh.harvard.edu/) based on a novel probabilistic atlas built using ex vivo brain MRI scans and histological data—technical details of which have been described in a previously published article [28] (Fig. 1). This atlas has been validated against earlier atlases, demonstrates excellent test-retest reliability, and is an improvement to previous atlases because of its probabilistic nature and ability to segment scans of arbitrary MRI contrasts [28]. Twenty-six subnuclei from each hemisphere were obtained for each participant. The 26 subnuclei were grouped into six major thalamic regions [28], namely the anterior, lateral, ventral, intralaminar, medial, and posterior nuclei. All thalamic segmentations were visually inspected for accuracy, and one participant with AD, from the original 66 recruited participants, was excluded because of inaccurate segmentation. Adopting a well-established definition of cerebral asymmetry [14], [17], [25], [32], [33], asymmetry between the right and left hemispheres was calculated for each participant as a lateralization index (LI) as such: LI = (L–R)/0.5 (L + R) * 100%, hence correcting for absolute bilateral volume of each respective structure. Accordingly, a positive LI denotes that the left hemisphere volume is larger than the right hemisphere volume, whereas a negative LI represents a larger right hemisphere volume compared with the left hemisphere volume.

### 2.2 Statistical analysis

Statistical analysis was performed using SPSS V21.0 (IBM SPSS Inc, Chicago, IL, USA). Normality of continuous data was tested using the Shapiro-Wilk test. Parametric data were analysed using either independent t-tests or analysis of variance, whereas nonparametric data were analysed using the Wilcoxon rank-sum test or Kruskal-Wallis test. Chi-square tests of independence were used for group comparisons of categorical variables. To compare absolute differences in thalamic subnuclei volume and the degree of subnuclei asymmetry between HC and participants with MCI and AD, analysis of covariance (ANCOVA) was conducted, controlling for age, gender, years of education, and total intracranial volume (TIV) to account for individual differences in head sizes. Multiple regression analysis, controlling for the same covariates, was conducted to investigate the associations of subnuclei asymmetry with clinical measures of cognition, neuropsychiatric symptoms, and functional ability.

To partial out the effects of inherent asymmetries of the whole brain and the hippocampus, additional analyses were conducted to include total grey matter asymmetry and hippocampal asymmetry as additional covariates in separate ANCOVA models (i.e., two separate ANCOVA models with covariates of gender, age, years of education, TIV, and an additional covariate of total grey matter asymmetry or hippocampal asymmetry) to avoid multicollinearity effects.

## 3 Results

### 3.1 Participant characteristics

Demographic and clinical characteristics of the participants are summarized in Table 1. The mean age of the whole cohort was 71.2 years (SD = 7.6), whereas the mean years of education was 14.1 years (SD = 3.1). The three groups were comparable in terms of gender (*P* = .814) but differed by age (*P* = .0495) and years of education (*P* = .027). Specifically, participants with MCI were significantly older (*P* = .020) and had fewer years of education (*P* = .008) than HC. As expected, HC scored higher than participants with MCI and AD on the MMSE (HC vs. MCI: *P* < .001; HC vs. AD: *P* < .001) and ACE-R (HC vs. MCI: *P* < .001; HC vs. AD: *P* < .001), although participants with MCI scored higher than those with AD (MMSE: *P* < .001; ACE-R: *P* = .008).

**Table 1:** Demographic characteristics of participants

	HC	MCI	AD	*P*-value
*N*	33	14	18	
Sex† *% Male*	54.5	50.0	61.1	.814
Age‡ *Mean in years(SD)*	69.0 (7.0)	74.6 (6.3)	72.3 (8.6)	.050*
Education§ *Mean in years (SD)*	14.9 (3.0)	12.3 (2.8)	13.9 (3.0)	.027*
MMSE§	28.9 (1.0)	25.9 (1.3)	24.3 (4.1)	<.001***
ACE-R§	93.0 (5.5)	80.6 (6.5)	72.8 (11.8)	<.001***
RAVLT§	44.0 (8.9)	29.2 (7.2)	20.5 (8.1)	<.001***
Verbal fluency§	11.6 (2.2)	10.1 (1.8)	7.0 (2.8)	<.001***
CDR¶	-	0.5 (0.2)	1.0 (0.5)	.002**
NPI¶	-	6.4 (8.7)	17.7 (18.1)	.009**
BADL¶	-	1.8 (2.1)	7.3 (7.3)	.002**

NOTE. Healthy controls were not assessed on CDR, NPI, and BADL.

Abbreviations: HC, healthy controls; MCI, mild cognitive impairment; AD, Alzheimer's disease; SD, standard deviation; MMSE, Mini-Mental State Examination; ACE-R, Addenbrooke's Cognitive Examination-Revised; RAVLT, Rey Auditory Verbal Learning Test; NPI, Neuropsychiatric Inventory; BADL, Bristol Activities of Daily Living.

*P < .05; ** *P* < .01; *** *P* < .001.

† Chi-square test of independence.

‡ Analysis of variance.

§ Kruskal-Wallis test.

¶ Mann-Whitney *U* test.

### 3.2 Comparisons of absolute thalamic subnuclei volumes

ANCOVA was conducted to compare absolute bilateral thalamic subnuclei volumes between the three diagnostic groups, controlling for gender, age, years of education, and TIV. The three groups differed on subnuclei volumes of the anterior (F [2,58] = 7.14, *P* = .002), lateral (F [2,58] = 4.79, *P* = .012), and posterior (F [2,58] = 9.03, *P* < .001) thalamus. On the other hand, no significant group differences were obtained for the whole thalamus (F [2,58] = 3.12, *P* = .052), or the ventral (F [2,58] = 1.21, *P* = .304), medial (F [2,58] = 2.35, *P* = .105), and intralaminar (F [2,58] = 0.51, *P* = .606) volumes. Post hoc tests showed that patients with AD had significantly smaller anterior and posterior thalamic volumes than HC (*P* = .001) and those with MCI (*P* = .008). Although the lateral and posterior nuclei were significantly smaller in patients with AD compared with HC (*P* = .004 and *P* < .001 respectively), these volumes were not significantly different between patients with AD and those with MCI. Anterior, lateral, and posterior thalamic volumes were comparable between HC and patients with MCI. The AD group also displayed smaller overall thalamic volume (*P* = .018) and medial thalamic volume (*P* = .036) compared with HC.

### 3.3 Comparisons of thalamic subnuclei asymmetry

**Fig. 2:**
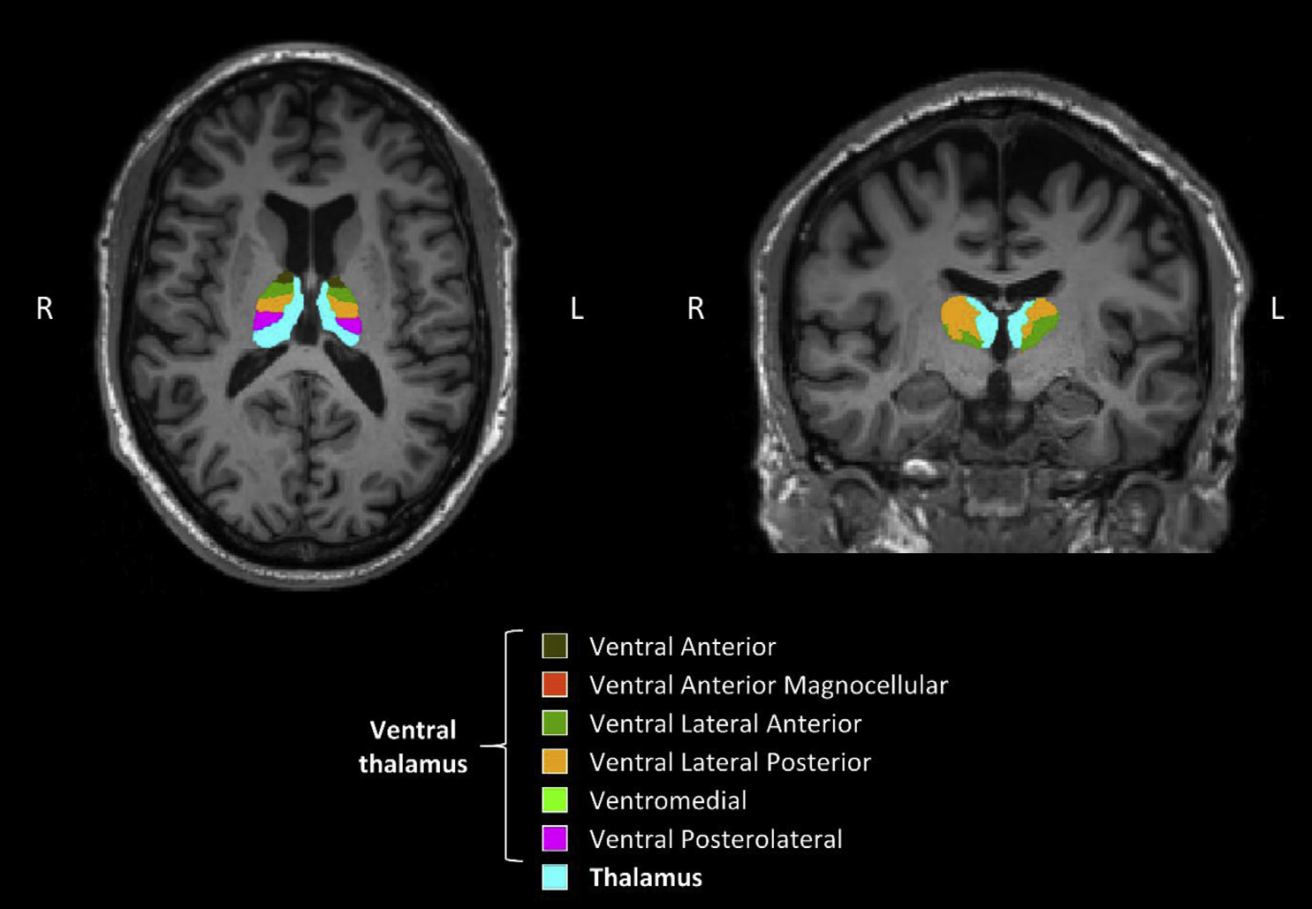
Asymmetrical ventral thalamus in a patient with Alzheimer's disease. Light blue represents the rest of the nonventral thalamus.

**Fig. 3:**
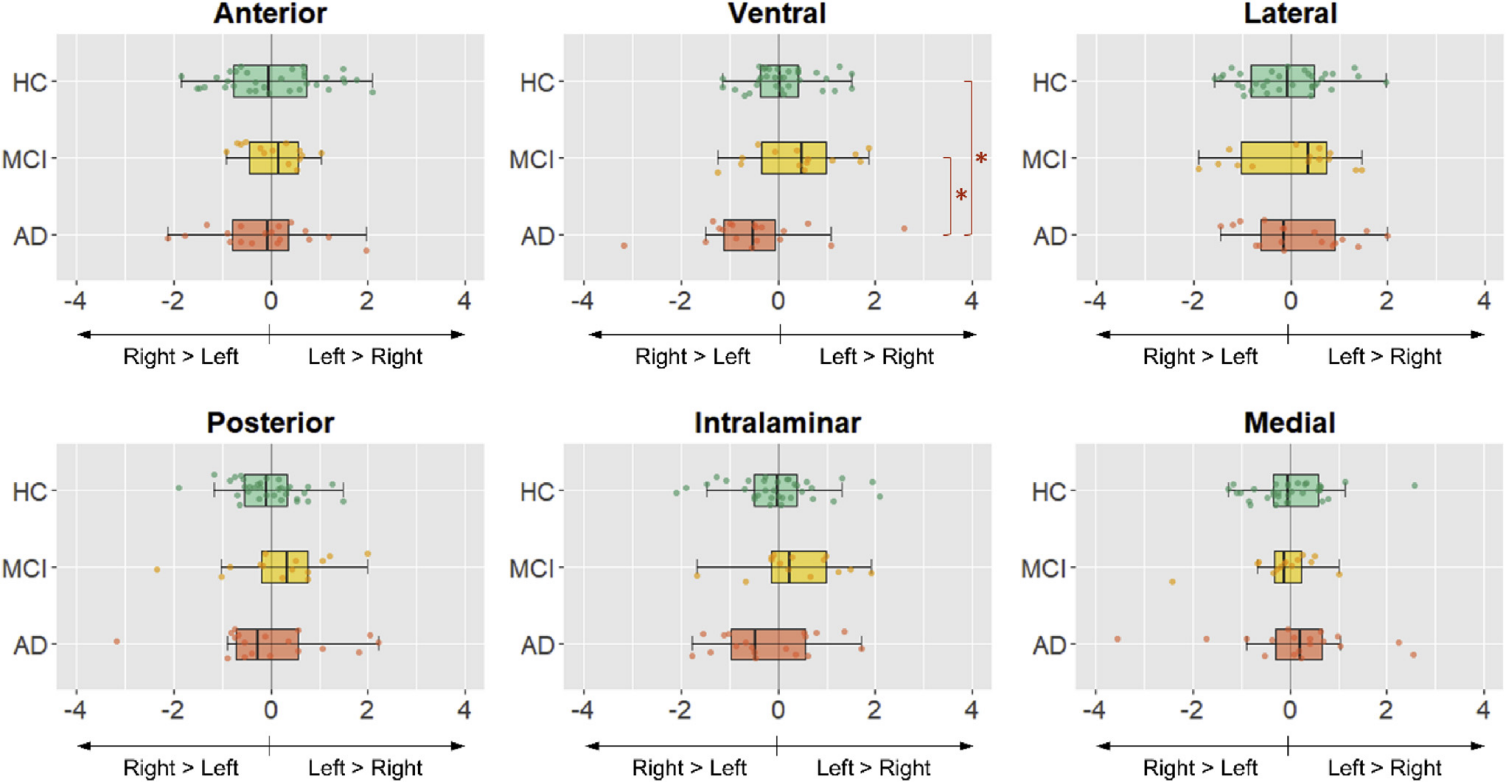
Boxplots comparing degree of thalamic subnuclei asymmetry in healthy controls (HC), mild cognitive impairment (MCI), and Alzheimer's disease (AD). Patients with AD displayed significantly smaller left than right ventral nuclei. The x-axis represents the laterality index (LI), wherein negative scores indicate greater right than left volume, and positive scores indicate greater left than right volume. LIs are residual-corrected for gender, age, years of education, and total intracranial volume (TIV). *Statistical significance at *P* < .05.

ANCOVA was conducted to compare the degree of subnuclei asymmetry between the three diagnostic groups, controlling for gender, age, years of education, and TIV. The three groups were comparable on overall thalamic asymmetry and most individual subnuclei but differed significantly on the degree of ventral nuclei asymmetry (F [2, 58] = 4.96, *P* = .010; Fig. 2 and 3). Post hoc analysis showed greater ventral thalamic asymmetry in patients with AD compared with those with MCI (*P* = .008) and HC (*P* = .012). Comparisons of the estimated marginal mean showed greatest R > L (i.e., greater volume in right hemisphere compared with left hemisphere) ventral asymmetry in patients with AD (mean = −6.32), compared with those with MCI (mean = −1.14) and HC (mean = −2.30; Fig. 1). The difference in ventral thalamic asymmetry between patients with MCI and HC did not reach statistical significance. Importantly, findings remained significant even after inclusion of total grey matter asymmetry (*P* = .021) and hippocampal asymmetry (*P* = .014) as additional covariates in separate ANCOVA models.

Given the significance of ventral thalamic asymmetry, we examined each subnuclei within the ventral region, namely the ventral anterior, ventral anterior magnocellular, ventral lateral anterior, ventral lateral posterior (VLP), ventral posterolateral (VPL), and ventromedial (VM) subnuclei. Within the ventral thalamus, group differences were restricted to specific subnuclei, namely the VM (F [2, 58] = 6.96, *P* = .002), VLP (F [2, 58] = 5.36, *P* = .007), and VPL (F [2, 58] = 3.81, *P* = .028) thalamic subnuclei. The significance of these contrasts remained after inclusion of total grey matter asymmetry or hippocampal asymmetry as additional covariates in separate analyses.

### 3.4 Clinical associations of thalamic subnuclei asymmetry

Multiple regression analysis was conducted to examine the associations of subnuclei asymmetry with clinical measures of disease severity, cognitive performance, and neuropsychiatric symptoms, controlling for gender, age, years of education, and TIV. To avoid the issue of range restriction, participants with MCI and AD were collectively grouped as cognitively impaired participants. Among these cognitively impaired participants, greater lateralization of leftward volume loss of the ventral nucleus was associated with poorer verbal fluency (β = .496, *P* = .007), higher Neuropsychiatric Inventory severity scores (β = −.394, *P* = .032), and marginally with a trend of higher CDR scores (β = −.377, *P* = .060) and lower total Rey Auditory Verbal Learning Test score (β = .371, *P* = .061) after adjusting for gender, age, years of education, and TIV. The degree of asymmetry in other thalamic nuclei was not associated with clinical measures.

**Fig. 4:**
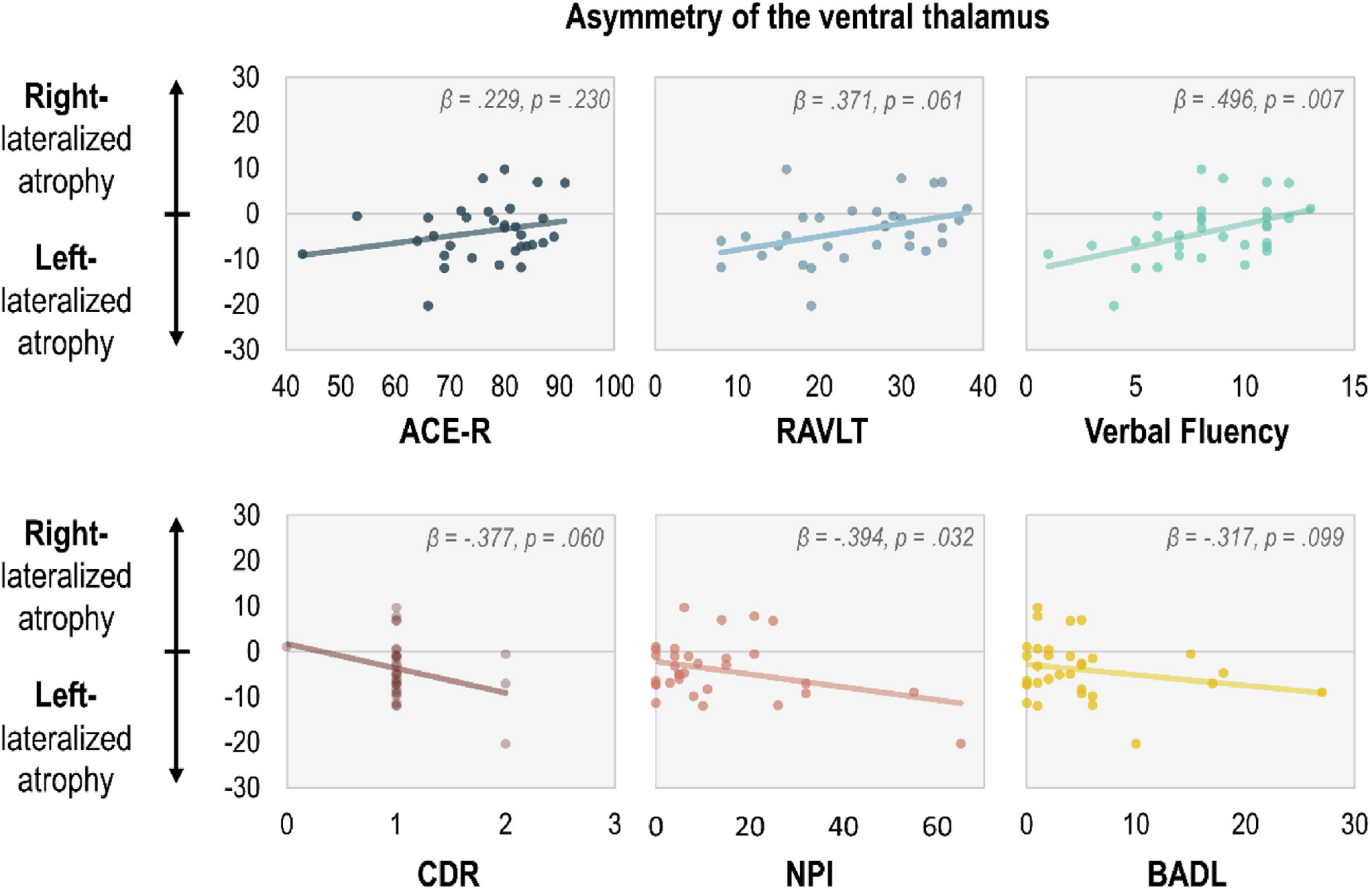
Scatterplots depicting the association between left-right asymmetry of the ventral thalamus on clinical measures. The y-axis represents the laterality index (LI) of the ventral thalamus. LIs are residuals corrected for gender, age, years of education, and total intracranial volume.

Examining the individual subnuclei of the ventral thalamus using multiple regression analysis (Table 2), we observed that performance on the ACE-R was only associated with lateralized leftward ventral thalamic damage of the VM, whereas no individual subnucleus was associated with the Rey Auditory Verbal Learning Test. Language fluency was associated with asymmetry of the VM, VPL, and VLP (Fig. 4). Associations with CDR scores were restricted to the VM and VPL subnuclei. In terms of neuropsychiatric symptoms, only VM and VPL subnuclei asymmetries were related to the Neuropsychiatric Inventory scores. With regard to functional mobility, only the left-lateralized damage of the VM and VPL subnuclei were related to the Bristol Activities of Daily Living score.

**Table 2:** Associations between clinical scores and asymmetry of individual ventral thalamic subnuclei in patients with mild cognitive impairment or Alzheimer's disease

Clinical measures	VAMC		VA		VLA		VLP		VPL		VM	
	β	*P*	β	*P*	β	*P*	β	*P*	β	*P*	β	*P*
ACE-R	0.40	.06	0.03	.88	0.09	.67	0.17	.38	0.34	.07	0.40	.03
RAVLT	−0.12	.59	0.32	.12	0.37	.07	0.31	.12	0.29	.14	0.26	.19
Verbal fluency	0.36	.09	0.35	.08	0.36	.07	0.44	.02	0.48	.01	0.55	<.01
CDR	0.11	.64	−0.19	.39	−0.25	.25	−0.34	.09	−0.42	.03	−0.50	.01
NPI	−0.15	.48	−0.21	.30	−0.24	.23	−0.29	.13	−0.49	.01	−0.46	.01
BADL	−0.33	.12	−0.08	.71	−0.16	.43	−0.31	.11	−0.38	.04	−0.54	<.01

NOTE. Multiple regression analysis controlling for gender, age, years of education, and TIV.

Abbreviations: VAMC, ventral anterior magnocellular; VA, ventral anterior; VLA, ventral lateral anterior; VLP, ventrolateral posterior; VPL, ventral posteriolateral; VM, ventromedial; ACE-R, Addenbrooke's Cognitive Examination–Revised; RAVLT, Rey Auditory Verbal Learning Test; NPI, Neuropsychiatric Inventory; BADL, Bristol Activities of Daily Living; TIV, total intracranial volume.

## 4 Discussion

The normal asymmetric structure and function of the human brain has evolutionary and developmental origins [34] but can also be indicative of neurological disorders [7], [34]. We examined the degree of thalamic asymmetry in AD, spanning amyloid-positive MCI and dementia. Thalamic atrophy was left lateralized in the ventral thalamus of patients with AD, specifically the VPL, VLP, and VM subnuclei, compared with HC and MCI groups. The left-lateralized atrophy of the posterior ventrolateral and VM nuclei correlated with verbal fluency, neuropsychiatric symptoms, disease severity, and functional impairment.

Our findings were consistent with previous investigations [5], [24], [25] in that patients with AD did not differ from nondemented participants on global thalamic asymmetries. A breakdown by subnuclei, however, revealed lateralization of atrophy. Although bilateral volumes of the ventral thalamus were not significantly different between groups, subnuclei analysis suggests that main effects may have been masked by the relative preservation of the right ventral thalamus in patients with AD, compared with the left ventral thalamus, which was disproportionately smaller in volume. Indeed, this left lateralization of ventral thalamic atrophy was more prominent in patients with AD, relative to nondemented participants. Owing to the fine level of segmentation afforded by the probabilistic atlas used, we were able to further examine if group differences were widespread across the ventral thalamus or localized to specific subnuclei, revealing that the left lateralization of thalamic atrophy in AD was largely restricted to the posterior ventrolateral and VM regions. Notably, these group differences were independent of the absolute subnuclei volume and global cerebral asymmetry and were significant even after accounting for hippocampal asymmetry which has been widely reported in AD [5], [12], [17].

Although overall thalamic asymmetry was not associated with global cognition, the leftward lateralization of ventral thalamic atrophy was associated with verbal fluency, independent of global cerebral asymmetry. This accords with the long-term language deficits following alterations to the left ventrolateral thalamus, but not the right, including impaired speech, and aphasic syndromes such as anomia, and reduced verbal output [35], [36], [37]. Accordingly, the ventral thalamus might play a role in coordinating the cognitive and motor aspects of language production [36].

The ventral thalamic changes also corresponded with neuropsychiatric symptoms. The involvement of the ventral thalamus in neuropsychiatric symptoms has been found in anxiety, panic disorders, and schizophrenia [38], [39]. In particular, schizophrenia has been shown to be associated with lesser neurons in the left ventrolateral posterior nucleus, but not the right [38]. Furthermore, general leftward cerebral atrophy has also been associated with delusions [8], apathy, and agitation [9] in AD, as well as post-traumatic stress disorder [40] and hallucinations [41]. Importantly, the associations with leftward ventral thalamic atrophy observed in this present study remained even after controlling for global brain asymmetry.

Among the small number of studies on the clinical associations of the ventral thalamus, most have focused specifically on the ventrolateral nucleus. However, owing to the fine thalamic segmentations used in this study [28], findings revealed that the involvement of the ventral thalamus in cognition and neuropsychiatric symptoms was localized to the posterior ventrolateral and VM nuclei of the left ventral thalamus (Fig 4). Although these thalamic regions are best known for their involvement in sensory/motor functions, they may also be involved in a greater complexity of function [42]. Furthermore, present findings support the position that distinct asymmetry of brain atrophy in AD may represent etiologically distinct subgroups [43], [44].

The reasons for thalamic asymmetries observed in this study are not known. One possibility involves the left-deficient asymmetry of norepinephrine in the ventral thalamus [45]. In a postmortem study, norepinephrine concentrations were strongly right lateralized in the VPL and ventral posteromedial nuclei, whereas in all other nuclei, concentrations were left lateralized [45]. Norepinephrine depletion has been linked to neuroinflammation and AD neuropathology [46] and may explain the leftward atrophy of the ventral thalamus. Conversely, the neuroprotective effects of norepinephrine may explain the relatively preserved right ventral thalamus [46]. Several suggestions have been made to explain the general left lateralization of cerebral damage in AD, including the increased sensitivity of the left hemisphere to stress [40], chronic hypoperfusion of the left hemisphere [47], and the increased propensity for hemodynamic stress and intimal damage in the left carotid artery [48]. Although these pathologies may account for global cerebral asymmetries, further research will be needed to establish the effects of these asymmetries on the thalamus.

The strengths of this study include the use of a probabilistic atlas based on ex vivo MRI scans and histological data for the segmentation of the thalamus, allowing estimates of subnuclei volumes. This probabilistic atlas may be used in combination with Bayesian inference to directly segment MRI of arbitrary contrasts. Furthermore, this study accounted for the bilateral volume of respective subnuclei, overall cerebral asymmetry, and hippocampal asymmetry, allowing us to determine that the effects observed in this study were independent of absolute subnuclei volumes and structural brain asymmetries, including that of the key structure affected in AD, the hippocampus. However, the cross-sectional design of this study prevents us from determining the mechanisms behind thalamic asymmetry and associated clinical measures. Moreover, because of the small sample size, findings should be replicated in a larger sample to validate our findings. To measure the degree of lateralization, we calculated the ratio of hemispheric difference in volume to mean bilateral volume, a widely accepted method of quantifying cerebral asymmetry [14], [17], [25], [32], [33]. Nevertheless, this method should be compared against other methods of measuring asymmetry [49]. Owing to the exploratory nature of this study, findings were not adjusted for multiple comparisons [50]. Although further a priori studies are required to confirm our findings, this study provides a reference for the clinical associations of thalamic subnuclei asymmetries to help guide the line of inquiry in subsequent investigations.

Overall, our study highlights the value of considering subregional cerebral asymmetries in clinical evaluations, and their relevance to clinical measures such as verbal fluency, functional ability, and neuropsychiatric symptoms. Further research should be conducted to investigate if the leftward ventral thalamic atrophy observed in this study is specific to AD and whether the measure of morphometric asymmetry could potentially be used to monitor disease progression and predict clinical outcomes.

**Research in Context**

1. Systematic review: Cerebral alterations in Alzheimer's disease are often asymmetric. This has been observed in terms of atrophy, microvascular changes, and amyloid-β and tau pathology. These asymmetries are associated with disease severity and poorer cognition. Although asymmetry exists across many structures (e.g., temporal lobe, hippocampus, amygdala, caudate), one structure of relevance in Alzheimer's disease that has not displayed the same asymmetries is the thalamus. Notably, such analyses have typically investigated the thalamus as a uniform structure.
2. Interpretation: This study revealed that thalamic asymmetries were confined to specific subnuclei in Alzheimer's disease, namely the ventral thalamic nuclei. Moreover, asymmetry of the ventral thalamus was associated with key clinical measures of verbal fluency, functional ability, and neuropsychiatric symptoms.
3. Future directions: The findings highlight the value of examining atrophy patterns at a subregional level. Further research should investigate if the leftward ventral thalamic atrophy observed in this study is specific to Alzheimer's disease.
